# Intergenerational Transfer of Tobacco Use Behaviour from Parent to Child: A Case Control Study

**DOI:** 10.31557/APJCP.2019.20.10.3029

**Published:** 2019

**Authors:** Chandrashekar Janakiram, Vinita Sanjeevan, Joe Joseph

**Affiliations:** *Department of Public Health Dentistry, Amrita School of Dentistry, Amrita University, Kerala, India. *

**Keywords:** Intergenerational, case control, smoking, smokeless Tobacco

## Abstract

**Background:**

Parental influence may be a strong modifiable risk factor in the initiation of Tobacco habits among young adults. Parenting style may modify the risk of initiation of Tobacco use.

**Objective:**

To examine the intergenerational transfer of Tobacco habits amongst the urban and tribal populations in Kerala.

**Methodology:**

A hospital based unmatched case control study was undertaken in urban and tribal health centres in Kerala, India. 239 cases (19-30 years of age using any form of Tobacco, 64.10% males) and 256 controls (35.90% males) were enrolled. Parental Tobacco exposure ascertainment was done by conducting in depth interviews using a validated structured questionnaire, parent bonding instrument and life grid technique. Multiple logistic regressions were performed.

**Results:**

The odds of a case initiating the habit of Tobacco use was nearly four times more when the parent was a Tobacco user [adjusted OR 4.26 (95% CI 2.39 – 7.58)] as opposed to controls. Among other covariates examined, low parental bonding with subject (especially father- warmth/care) was a strong risk factor for Tobacco usage [OR 2.17 (95% 1.11 – 4.23)]. The cases had nearly four times the probability of Tobacco uptake compared to controls if the mothers had no formal schooling [adjusted OR of 3.93 (95% CI, 2.12 – 7.26)].

**Conclusion:**

Parental use of Tobacco influences the uptake of Tobacco habits in their children, with the father’s parenting (low paternal warmth) being a strong risk factor.

## Introduction

The initiation of Tobacco use in young adults is influenced by cultural and social factors (Conrad et al., 1992). Significant among these are parent to child transmission, pressure from peers and perceived benefits of smoking (Talip et al., 2016). It is very unlikely that an individual will initiate the use of Tobacco if the habit doesn’t start during adolescence (Tyas and Pederson, 1998) During this period, the intergenerational (parent to child) transfer of habits is an important contributor for the initiation of Tobacco use. The Social Learning theory by Bandura emphasizes that people with whom one regularly associates, delimits the types of behaviour that one will repeatedly observe and hence learn, which explains reasons for intergenerational transfer and influences from peers (Johnston et al., 2012; Subramaniam et al., 2015). 

 In rural Indian communities, family members often seek help from children to purchase chewable Tobacco products from stores (Kakde et al., 2012). This trigger in early formative years, may be responsible for the child to perceive Tobacco use as acceptable. Additionally, when parents (either both or single) themselves are Tobacco users, attempts to impose restriction in their children may not be effective due to credibility issues (Holdsworth and Robinson, 2013). Casual mentions of Tobacco in the household may further, trigger curiosity (Bantle and Haisken-DeNew, 2002). 

Parenting style has a modifying effect on the transfer of Tobacco habits between generations. Culture serves as a guiding framework to parents while rearing their children (Londhe, 2015). The strong protective or formal parenting style in Indian society differs from the western culture of fostering independence. 

The intergenerational transfer of the Tobacco habit has been studied in developed countries (Bantle and Haisken-DeNew, 2002; El-Amin et al., 2015; Escario and Wilkinson, 2015; Gilman et al., 2009; Göhlmann et al., 2010; Leonardi-Bee et al., 2011; Mahabee-Gittens et al., 2012; Mays et al., 2014; Melchior et al., 2010; Vuolo and Staff, 2013; White et al., 2000) but scarcely reported in southeast Asian countries (Madathil et al., 2015), where smokeless Tobacco (SLT) consumption is high. We designed this study to assess if the parental Tobacco use behaviour is a risk factor for initiation of Tobacco use among their children Additionally, we aimed to understand the effect of parenting style on the transfer of such behaviours. We chose to look at Tobacco use irrespective of the form it is consumed in (smoked/smokeless), as the risk posed with its use and transfer across generations remains unchanged.

## Materials and Methods

*Study design*

**Figure 1:**
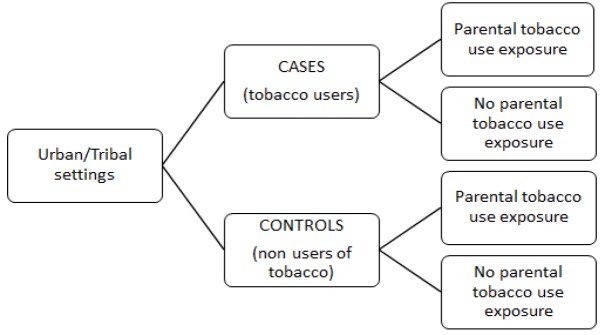
Case Control Design

We designed this hospital-based case-control study to compare the exposure distribution (parental Tobacco use) between groups of Tobacco users and non-users as given in Figure 1. 

*Study Setting*

We identified two healthcare facilities for this study; an urban centre (Cochin) and a tribal centre (Kalpetta) Kerala, India.

*Case and Control Selection*

1. Individuals visiting the health centre as bystanders of patients (individuals accompanying the patient to the health facility and not requiring consultation or treatment themselves on the concerned day) were identified. These individuals were interviewed for their Tobacco usage status and were classified as: 

A. Cases if they used any form of Tobacco; chewing or smoking or both. 

B. Controls if they had never used any form of Tobacco in their lifetime 

2. Participants were aged between 19 – 30 years.

3. All participants were required to be continuous life residents of the concerned area (urban/tribal) and should have been raised by their parents until 18 years of age. [Continuous life residents are those who are born, reared and living in the same area except for a few weeks (holidays) in the year]. 

The study was approved by institutional ethics board of Amrita Institute of Medical Sciences (Ref/011/TPRC/2016). A written informed consent was obtained from all volunteering participants. This research was conducted in full accordance with the World Medical Association Declaration of Helsinki. The data collection period extended from May 2016 to October 2017. The research instrument was pilot tested in 2016 and modifications were made accordingly.

*Data Collection*

Data collection was done in two stages; 

1. Identification of cases and controls by trained research assistants. 

2. Investigator (VS) ascertained the parental Tobacco history using the research instrument, facilitated further using life grid technique. 

To avoid selection bias and ascertainment bias, the investigator (VS) was blinded for Tobacco status of the case and control. 

*Exposure Ascertainment*

To ascertain the parental Tobacco exposure, we administered a structured validated questionnaire to interviewees’ (cases and controls) uniformly. The content validity of the questionnaire was done by three experts in the field of psychology using Content Validity Index (CVI). CVI score was found to be 0.95. Information such as parent’s demographics, their Tobacco use behaviour (frequency, duration etc.), educational status, socioeconomic status was collected. 

*Life grid technique*

We employed the life grid technique to facilitate recall of parental Tobacco history from cases and controls. This technique works on the principle that recalled information on certain social circumstances when cross-referenced with the information sought for the study, provides a useful degree of accuracy, minimizing recall bias. Besides controlling recall bias, the process of going through the participants’ life events in the form of a life grid, helped us establish a positive rapport with the participants, which in turn allowed easier elicitation of sensitive information (Berney and Blane, 1997; Blane, 1996). 

**Table 1:** Characteristics of the Population

Variables	Cases (n)	%	Controls (n)	%	Total N (%)	Odds ratio [CI 95%]
Age						
19 – 24 years	151	45.07	184	54.92	335 (67.67)	0.67
25 – 30 years	88	55	72	45	160 (32.52)	[0.46 – 0.98]
Gender						
Male	134	64.11	75	35.88	209 (42.22)	3.08
Female	105	36.71	181	63.28	286 (57.77)	[2.12 – 4.46]
Location						
Urban	113	46.12	132	53.87	245 (49.50)	0.84
Tribal	126	50.4	124	49.6	250 (50.50)	[0.59 – 1.19]
Religion						
Hinduism	200	50.63	195	49.36	395 (79.79)	1.6
Others	39	39	61	61	100 (20.20)	[1.02 – 2.51]
Marital status						
Unmarried	144	46.75	164	53.24	308 (62.22)	0.85
Married	95	50.8	92	49.19	187 (37.77)	[0.59 – 1.22]
Educational status						
Up to high school	164	51.89	152	48.1	316 (63.83)	1.49
Diploma and higher	75	41.89	104	58.1	179 (36.16)	[1.03 – 2.16]
Occupation						
Unemployed	150	42.73	201	57.26	351 (70.90)	0.46
Employed	89	61.8	55	38.19	144 (29.09)	[0.31 – 0.68]
Fathers education						
Up to high school	227	51.12	217	48.87	444 (89.69)	3.4
Diploma and higher	12	23.52	39	76.47	51 (10.30)	[1.73 – 6.66]
Mothers education						
Illiterate	96	65.75	50	34.24	146 (29.49)	2.76
Educated	143	40.97	206	59.02	349 (70.50)	[1.84 – 4.13]

**Table 2:** Distribution of Exposure - Parents’ Tobacco Use Status among Cases and Controls

Parents Tobacco use	Cases (Tobacco))		Controls		Total		Odds Ratio
	n	%	n	%	N	%	(95% CI)
User parent	202	59.06	140	40.93	342	100	4.52 [2.94 – 6.94]
Non-user parent	37	24.18	116	75.81	153	100	1
Total	239	48.28	256	51.71	495	100	
	Cases (smokers)		Controls		Total		Odds Ratio
	n	%	n	%	N	%	(95% CI)
Smoker parents	69	56.55	53	43.44	122	100	2.79 (1.85 – 4.19)
Non-user parents	146	39.89	313	85.51	366	100	
Total	215	37	366	62.99	581	100	
Chewer parent	5	0.03	143	96.62	148	100	0.07 (0.03 – 0.18)
Non-user parent	146	31.8	313	68.19	459	100	
Total	151	24.87	456	75.12	607	100	
Both form parent	0	0	3	100	3	100	0.7 (0.07 – 6.92) *
Non-user parent	146	31.8	313	68.19	459	100	
Total	146	31.6	316	68.39	462	100	
	Cases (chewers)		Controls		Total		Odds Ratio
	n	%	n	%	N	%	(95% CI)
Chewer parents	238	62.46	143	37.53	381	100	34.72 (19.87-60.67)
Non-user parents	15	4.57	313	95.42	328	100	
Total	253	35.68	456	64.31	709	100	
Smoker parent	1	1.85	53	98.14	54	100	0.39 (0.05 – 3.04)
Non-user parent	15	4.57	313	95.42	328	100	
Total	16	4.18	366	95.81	382	100	
Both form parent	4	7.01	53	92.98	57	100	1.57 (0.50 – 4.92)
Non-user parent	15	4.57	313	95.42	328	100	
Total	19	4.93	366	95.06	385	100	

* Yates correction performed

**Table 3:** Multivariate Analysis

Variable	Reference	Unadjusted Odds Ratio	95% Confidence Interval		Odds Ratio	95% Confidence Interval	
			Lower	Upper		Lower	Upper
Age	19 - 24 years	0.67	0.46	0.98	0.9	0.52	1.53
Gender	Male	3.08	2.12	4.46	9.39	4.64	18.99
Location	Tribal	0.84	0.59	1.19	0.58	0.26	1.3
Religion	Hinduism	1.6	1.02	2.51	1.14	0.59	2.21
Marital status	Un-Married	0.85	0.59	1.22	0.85	0.46	1.55
Education	Up to High school	1.49	1.03	2.16	1.53	0.83	2.84
Occupation	Un-Employed	0.46	0.31	0.68	0.99	0.57	1.73
Father education	Up to High school	3.4	1.73	6.66	2.01	0.83	4.87
Mothers education	Illiterate	2.76	1.84	4.13	3.93	2.12	7.26
Fathers occupation	Un-Employed	1.82	1.01	3.28	1.23	0.56	2.71
Mothers occupation	Un-Employed	0.77	0.54	1.1	1.47	0.89	2.42
Fathers warmth	High Warmth	3.16	1.94	5.17	2.17	1.11	4.23
Mothers warmth	Low Warmth	0.8	0.5	1.26	0.52	0.27	1.00
Fathers protection	High Protection	1.68	1.11	2.54	1.03	0.46	2.30
Mothers protection	High Protection	2.4	1.55	3.71	2.19	0.99	4.86
Parents Tobacco use	User	4.52	2.94	6.94	4.26	2.39	7.58

*Parent bonding instrument (PBI)*

We used PBI, a validated and reliable scale to measure fundamental parental styles as perceived by the cases and controls about their parents which was categorised as; ‘*warmth/support/care*’ and ‘*protection/control/demandingness*’. The measure was retrospective, meaning that the participants completed the measure for how they remembered their parental role towards them during their first 16 years. The measure was to be completed for either parents separately. This 25-item questionnaire consisted of questions related to caring (12 questions) and warmth attitudes (13 questions). Each item was scored to generate a care and protection score for each of the parent. Predefined cut off scores enabled classification of the parenting style as high or low caregiving and high or low levels of controlling. The cut-off score for ‘care’ was 24 and 27 for fathers and mothers respectively. Scoring above the cut-off value deemed the parent as high care giving while scores below as low care giving. Cut-off scores for the ‘control’ was 12.5 and 13.5 for fathers and mothers respectively. Scores above these cut-off were deemed as high levels of controlling (Parker et al., 1979).

*Sample size estimation*

Using estimates from previous studies, prevalence of smoking Tobacco (0.18 control and 0.12 cases)(Melchior et al., 2010) and chewing Tobacco (0.28 control and 0.61 cases)(Madathil et al., 2015) were obtained and we estimated a sample size of 418 (209 each for cases and controls) with power 80% and alpha error of 0.5. The final sample size was rounded to 250 each for cases and control to cover attrition and other covariates. 

*Statistical analysis*

The data was processed using Statistical Package for Social Sciences (SPSS, IBM Version 20). Initially, a series of bivariate analysis were carried out, followed by a stratified analysis of selected variables. Multivariate logistic regression models were used to estimate the odds ratios and the associated 95% confidence intervals (CI). 

## Results

Descriptive characteristics of cases and controls are given in Table 1. Since, it was an unmatched case–control study, there were variations in the distribution of cases and controls in terms of characteristics. A total of 495 participants; 239 cases and 256 controls were enrolled in this study with 49.6% of the participants belonging to the tribal setting. The participants were aged between 19 and 30 years (57.77% females and mean age ± standard deviation of 23.16 ± 3.53 years). 63.83% participants had an ‘up to high school’ level of education and 70.9% of the participants were not employed. 84.51% and 54.68% of cases and controls respectively had Tobacco user parents. (Table 2). Among the 113 cases in the urban area 97.34% used the smoked form, while in the tribal area all 126 (100%) cases used the smokeless form of Tobacco.

Stratified analysis given in table 2 showed that when parents were smokers, cases were three times more likely to smoke as opposed to controls [odds ratios 2.79 (95% CI, 1.85 – 4.19)]. When parents were users of smokeless Tobacco the likelihood of a cases using it increases exponentially to 35 times as much as controls of smokeless Tobacco user parents [OR 34.72 (95% CI, 19.87 – 60.67)]. However, we found that it was unlikely that participants picked up the habit of smoking, if their parents were users of smokeless Tobacco when compared to controls [OR 0.07 (95% CI, 0.03 – 0.18)].

The multivariate model provided in table 3, showed that cases were four times more likely to use Tobacco compared to controls if their parents had a history of Tobacco use (adjusted odds ratio of 4.26 (95% 2.39 – 7.58)). The cases had a nearly four times probability of Tobacco uptake compared to controls if the mothers had no formal schooling [adjusted OR of 3.93 (95% CI, 2.12 – 7.26)]. The cases whose fathers exhibited low warmth towards them were at nearly two times risk of taking up Tobacco use habit compared to controls OR 2.17 (95% CI, 1.11 – 4.23). Males had a nine times risk [adjusted OR 9.39 (95% CI, 4.64 – 18.99) of taking up the habit as compared to females. 

## Discussion

We found a strong association between parental Tobacco use and the uptake of the same among their children. Cases are likely to smoke or chew nearly four times more when their parents had a history of Tobacco use as opposed to controls. This intergenerational transfer has been studied in western societies (Madathil et al., 2015; Mahabee-Gittens et al., 2012; Vandewater et al., 2014; Vuolo and Staff, 2013; White et al., 2000) and similar strong associations have been found. The findings of our study show the risk of transfer of smoking habits from a parent to a smoker case to be nearly three times when compared to a control [OR 2.79 (95%CI 1.85 – 4.19. Our findings were consistent with previous studies by Vandewater et al., (2014), Melchoir et al., (2010), and Diwedi et al., (2016) that presented odds of child’s smoking given a parent was smoker as 2.91 (1.60 – 5.31), 1.96 (1.30 – 2.79) and 3.47 (2.17-5.53) respectively. The risk of intergenerational transfer increases exponentially to 35 times for smokeless Tobacco, if cases had parental history of smokeless Tobacco use as compared to controls. This may be attributed to the fact that all cases from tribal areas were using smokeless Tobacco which is an accepted social norm as compared to mainland urban areas (Janakiram et al., 2016; Valsan et al., 2016). However, it was unlikely to find a smoker case when parents were Tobacco chewers, highlighting a form-specific transfer of Tobacco use behaviour between generations.

These findings reaffirm the concept of social learning theory that children mould their behaviour using their parental behaviour as example. Children observe parents at close quarters, and habits practiced by them are oftentimes perceived as appropriate. Whereas, direct stimulus for Tobacco initiation may be from sources like peers, stress, boredom etc; the trigger may arise from the deep-rooted internalisation of the parent’s behaviour. This is reaffirmed by the description of stages of smoking among adolescents by Mayhew et al., (2000). Males had a nine times risk of taking up the habit as compared to females. In India, Tobacco use is predominantly a male behaviour, particularly the smoked form. These findings are coherent to cultural effect in India wherein prevalence of smoking Tobacco is high in males in contrast to smokeless Tobacco in females (Bhawna, 2013). 

When the paternal care attitude is high, the likelihood of the cases taking up the habit is reduced. This study was suggestive that father’s care could provide a protective influence on the cases and hence prevent the initiation of Tobacco use. This finding is consistent with other studies. In a study by Gittens et al., (2009) it has been shown that increased parental monitoring is associated with decreased odds of smoking initiation (33%) while decreased parental monitoring is associated with increased odds of smoking in children (55%). So also, in a critical review of literature on the psychosocial factors related to adolescent smoking, Tyas et al., (1998) report that an authoritative positive parenting style is associated with lower levels of adolescent smoking and that low parental concern increases the risk of uptake of smoking among boys. It has been shown from results of this study that the child attempts to imitate the parent of the same gender. Parental warmth has been associated with decrease in externalizing behaviours such as alcohol consumption and increase in self-esteem of the adolescent (Rosenberg and Wilcox, 2006). This finding has been seen consistently across all ethnic groups (Hoskins, 2014). Madathil et al., (2015) concluded maternal strictness was associated with decreased Tobacco uptake by the child. Though our study findings are distinct from the previous study, it is of substantial significance, as unlike the traditional concept of mother’s central role in the rearing of a child; our study results highlight the father’s role in the process of parenting. 

Mother’s education had a significant role to play in the initiation of Tobacco habits by the child. A study on the parental education and family status’s association with children’s cigarette smoking concluded that higher education of the mothers significantly lowered the frequency of current experimentation and decision about future smoking among children (Zaloudíková et al., 2012).This is relevant as it has been proven that the mother’s education has a crucial role to determining the health behaviours of a family (Tyas and Pederson, 1998).

*Strengths and limitations*

This study has several strengths and some limitations. Cases and controls were selected from the same study base, comparable areas and participation was complete, thus reducing the likelihood of selection bias. The bystanders of patients who had visited for other reasons (non-Tobacco) were enrolled, thereby decreasing bias as the researchers had no access to the participant’s medical history, hence no knowledge of the Tobacco status prior to enrolment. Recall bias was controlled to a large extent using the life grid technique, which also proved to be beneficial in overcoming information bias. Blinding the principal investigator to the outcome helped overcome interviewer’s bias. However, bias arising from non-response of participants and diagnostic suspicion bias could not be controlled.

Limitations in the form of confounders like peer pressure also need to be mentioned here. It was beyond the scope of this study to adjust for this and hence was not chosen to be included in the study question. It is necessary, here to mention that parental transmission is not the only factor influencing a child’s inclination to explore Tobacco, several other factors have an interplay in this, however, parents are a constant element in a child’s life, unlike peers who may change over years.

*Policy implications*

Adolescent population is one group that may easily succumb to the use of Tobacco. The argument for Tobacco use prevention among adolescents is based on the observation that if Tobacco use does not start during adolescence, it is unlikely ever to occur.

Hence, public health programs have aggressively targeted this population (Tyas and Pederson, 1998). Most of the efforts have been directed at young adults in an individualistic approach. We propose here, the need to focus on families rather than individuals. There is a need to advocate for Tobacco-free homes campaigns at schools. There is evidence that engaging parents in such school-based anti-Tobacco campaigns make them more effective (Murray et al., 1985). Second and third-hand smoking harms have been projected extensively to curb Tobacco use among parents. Important though this approach is, there is also a need to educate parents on the possibility of their child initiating Tobacco, merely by mirroring parental Tobacco use behaviour. This knowledge gives parents the opportunity to make corrections to their own Tobacco use behaviour, in turn ensuring a Tobacco-free home. One needs to focus on the finding that adequate care or support from the parent may defer the child from picking up the habit, reinforcing the benefits of right parenting.

Mothers are believed to be primary caregivers. Maternal education is considered to be related to health behaviours in a household (Tyas and Pederson, 1998). Hence, equipping women with knowledge is mandated.

Finally, our study has shown a strong tendency for male offspring to take up the habit. Here it is noteworthy that this trend is soon changing, and efforts must be targeted at both genders.

In conclusion, a child’s Tobacco initiation is strongly associated with the parent’s Tobacco use behaviour. However, father’s warmth towards his child and the mother’s educational status both, have a modifying effect on the uptake of this behaviour. Overall the transfer of Tobacco habits across one generation, particularly among the male child was high when parents used Tobacco, and this calls for concerted efforts in making parents take responsibility for their actions and child health.

## Declaration

Ethics approval and consent to participate. The study was approved by institutional ethics board of Amrita Institute of Medical Sciences (Ref/011/TPRC/2016). A written informed consent was obtained from all volunteering participants. This research was conducted in full accordance with the World Medical Association Declaration of Helsinki.
